# Supplementary material for: Neutrophils direct preexisting matrix to initiate repair in damaged tissues
Source: Nat Immunol. 2022 Mar 30;23(4):518–31. doi: 10.1038/s41590-022-01166-6 (PMC8986538; doi:10.1038/s41590-022-01166-6)
Supplement: Supplementary file 1 — Reporting Summary [file 41590_2022_1166_MOESM1_ESM.pdf]

nature portfolio

Corresponding author(s): Yuval Rinkevich

Last updated by author(s): Feb 17, 2022

Reporting Summary

Nature Portfolio wishes to improve the reproducibility of the work that we publish. This form provides structure for consistency and transparency in reporting. For further information on Nature Portfolio policies, see our Editorial Policies and the Editorial Policy Checklist.

Statistics

For all statistical analyses, confirm that the following items are present in the figure legend, table legend, main text, or Methods section.

n/a	Confirmed	
☐	☒	The exact sample size (*n*) for each experimental group/condition, given as a discrete number and unit of measurement
☐	☒	A statement on whether measurements were taken from distinct samples or whether the same sample was measured repeatedly
☐	☒	The statistical test(s) used AND whether they are one- or two-sided<br>*Only common tests should be described solely by name; describe more complex techniques in the Methods section.*
☒	☐	A description of all covariates tested
☒	☐	A description of any assumptions or corrections, such as tests of normality and adjustment for multiple comparisons
☐	☒	A full description of the statistical parameters including central tendency (e.g. means) or other basic estimates (e.g. regression coefficient) AND variation (e.g. standard deviation) or associated estimates of uncertainty (e.g. confidence intervals)
☐	☒	For null hypothesis testing, the test statistic (e.g. *F*, *t*, *r*) with confidence intervals, effect sizes, degrees of freedom and *P* value noted<br>*Give P values as exact values whenever suitable.*
☒	☐	For Bayesian analysis, information on the choice of priors and Markov chain Monte Carlo settings
☒	☐	For hierarchical and complex designs, identification of the appropriate level for tests and full reporting of outcomes
☒	☐	Estimates of effect sizes (e.g. Cohen's *d*, Pearson's *r*), indicating how they were calculated

Our web collection on statistics for biologists contains articles on many of the points above.

Software and code

Policy information about availability of computer code

Data collection	Leica Application Suite v4.8<br>AxioVision (Carl Zeiss) v4.8<br>ZEN2009 (Carl Zeiss) v.5.5<br>Progenesis QIsoftware (version 4.1, Nonlinear Dynamics, Waters)<br>BD FACSDIVA v9.0
Data analysis	ImageJ (1.52i)<br>Imaris (v9.1.0)<br>Mascot (Matrix Science, version 2.6.2)<br>GraphPad Prism (v.7.0, GraphPad)<br>flowjo 10.7.

For manuscripts utilizing custom algorithms or software that are central to the research but not yet described in published literature, software must be made available to editors and reviewers. We strongly encourage code deposition in a community repository (e.g. GitHub). See the Nature Portfolio guidelines for submitting code & software for further information.

Data

Policy information about availability of data

All manuscripts must include a data availability statement. This statement should provide the following information, where applicable:

- Accession codes, unique identifiers, or web links for publicly available datasets
- A description of any restrictions on data availability
- For clinical datasets or third party data, please ensure that the statement adheres to our policy

Data is in respective Source file.
The proteomics data are deposited at PRIDE under submission ID: px-submission #564638.
The scRNA-seq data are accessible to all. The raw files will be added to a public repository in the next 3-4 days.
Computer Code is available at: https://github.com/kadrisaf/Fluid_Matrix

Field-specific reporting

Please select the one below that is the best fit for your research. If you are not sure, read the appropriate sections before making your selection.

☒ Life sciences ☐ Behavioural & social sciences ☐ Ecological, evolutionary & environmental sciences

For a reference copy of the document with all sections, see nature.com/documents/nr-reporting-summary-flat.pdf

Life sciences study design

All studies must disclose on these points even when the disclosure is negative.

Sample size	No statistical method was used to predetermine sample size. Required experimental sizes were estimated based on previous established protocols in the field (e.g. DOI: 10.1038/s41586-019-1794-y,). The sample sizes were adequate as the differences between experimental groups were reproducible and were statistically significant, demonstrating the suitability of the sample size. All n values are clearly indicated within the figure legends.
Data exclusions	No data was excluded from the analysis.
Replication	All experiments were performed at least three times with similar results.
Randomization	Mice and ex vivo tissues were randomly divided into treatment groups.
Blinding	No experiments presented in this study required blinding. Critical in vivo adhesion scorings were on-off effects in all replicates. Samples were measured using automated methods across different assays with different personnel, which reduces the subjectivity to a justifiable minimum.

Reporting for specific materials, systems and methods

We require information from authors about some types of materials, experimental systems and methods used in many studies. Here, indicate whether each material, system or method listed is relevant to your study. If you are not sure if a list item applies to your research, read the appropriate section before selecting a response.

Materials & experimental systems

n/a	Involved in the study	
☐	☒	Antibodies
☒	☐	Eukaryotic cell lines
☒	☐	Palaeontology and archaeology
☐	☒	Animals and other organisms
☐	☒	Human research participants
☒	☐	Clinical data
☒	☐	Dual use research of concern

Methods

n/a	Involved in the study	
☒	☐	ChIP-seq
☐	☒	Flow cytometry
☒	☐	MRI-based neuroimaging

Antibodies

Antibodies used	Primary antibodies:<br>COLLAGEN I (Rockland, 600-401-103-0.1, 1:150)<br>COLLAGEN VI (Abcam, ab6588, 1:150)<br>Fibronectin (Abcam, ab23750, 1:100)<br>HSPG2 (Elabscience, E-AB-13507.20, 1:100),

cleaved Caspase 3 (Abcam, 9661S, 1:100),
Laminin (Abcam, ab11575, 1:100),
PDPN (Abcam, ab11936, 1:100),
LY6G (Abcam, ab25377, 1:100),
F4/80 (Abcam, ab90247, 1:400),
TNF (Abcam, ab223352, 1:150),
ITGAM (Abcam, ab8878, 1:100),
ITG[illegible]2 (Abcam, ab63388, 1:150),
CD45.1 (Abcam, ab23910, 1:150),
FPR1 (Abcam, ab113531, 1:100),
CD62L (Abcam, ab119834, 1:100),
YAP/TAZ, (Abcam, ab205270, 1:100),
PDGFRA (R&D systems, AF1062, 1:50) ,
phosphoSMAD2/3 (Abcam, 18338S, 1:100),
phosphoHSF (Elabscience, E-AB-20894.60, 1:100),
Ncadherin (Abcam, ab18203, 1:100),
GAPDH (Life Scientist, MA515738, 1:1000).
Kindlin3 (Sigma, PRS4797,1:1000)

FACS:
Reagent or Antibodies Cat. Co.
APC anti-mouse CD45 , Rat IgG2b, kappa, Clone: 30-F11 100 µg 103112 Biolegend
Pacific Blue™ anti-mouse Ly-6G 100 µg 127612 Biolegend

Following products (1:500, Life technologies) against suitable species were used as secondary antibodies.
Alexa Fluor 488 Donkey Anti-Rabbit (A21206),
Alexa Fluor 594 Donkey Anti-Rabbit, (A21207),
Alexa Fluor 594 Donkey Anti-Goat, (A32758),
Alexa Fluor 647 Donkey Anti-Goat, (A-21447),
Alexa Fluor 647 Donkey Anti-Rabbit IgG, (A31573),
Alexa Fluor 647 Goat Anti-Rat, (A21247).

Validation

All antibodies have been validated by the manufacturer and by multiple citations for reactivity against mouse or human.
Abcam: “We use a variety of methods, including staining multi-normal human tissue microarrays (TMAs), multi-tumor human TMAs, and rat or mouse TMAs during antibody development. These high-throughput arrays allow us to check many tissues at the same time, providing uniformly as all tissues are exposed to the exact same conditions. “

Antibodies were additionally validated using respective isotype antibodies in immunofluorescence assays. Negative controls were performed in all staining procedures.

Animals and other organisms

Policy information about studies involving animals; ARRIVE guidelines recommended for reporting animal research

Laboratory animals

Following mouse strains were used, both males and females, adult at 8-12 weels old:
C57BL/6J wild type, B6.129P2-Lyz2tm1(cre)Ifo/J (Lyz2Cre), B6;129S6-Gt(ROSA)26Sortm14(CAG-tdTomato)Hze/J (Ai14), En1Cre (En1tm2(cre)Wrst), En1Cre (En1tm2(cre)Wrst) and R26mTmG (Gt(ROSA)26Sortm4(ACTB-tdTomato,-EGFP)Luo) mice were purchased from Jackson Laboratories or Charles River, R26CreER;floxKindlin3flox mice were a gift from R.F. and P.K.. All mice were bred and maintained in the Helmholtz Animal Facility in accordance with EU directive 2010/63. Animals were housed in individual ventilated cages and animal housing rooms were maintained at constant temperature at 20-24 °C and humidity at 45-65% with a 12 hours light cycle. Animals were supplied with water and chow ad libitum. All animal experiments were reviewed and approved by the Government of Upper Bavaria and conducted under strict governmental and international guidelines. This study is compliant with all relevant ethical regulations regarding animal research.

Wild animals

This study did not involve wild animals.

Field-collected samples

The study did not involve samples collected from field.

Ethics oversight

Government of Upper Bavaria, Germany.

Note that full information on the approval of the study protocol must also be provided in the manuscript.

Human research participants

Policy information about studies involving human research participants

Population characteristics	Patients undergoing mechanic adhesiolysis between 50-65 years of age, both genders.
Recruitment	All human samples were obtained from surgery at the Department of Surgery, Klinikum rechts der Isar, Technical University of Munich. Adhesions were intraoperatively diagnosed and dissected from the respective organs and prepared for further analysis.
Ethics oversight	Approval of the local ethics committee of the Technical University of Munich, Germany (Nr. 173/18 S).

Note that full information on the approval of the study protocol must also be provided in the manuscript.

Flow Cytometry

Plots

Confirm that:

☒ The axis labels state the marker and fluorochrome used (e.g. CD4-FITC).

☒ The axis scales are clearly visible. Include numbers along axes only for bottom left plot of group (a 'group' is an analysis of identical markers).

☒ All plots are contour plots with outliers or pseudocolor plots.

☒ A numerical value for number of cells or percentage (with statistics) is provided.

Methodology

Sample preparation	Murine blood were collected by cardiac puncture and diluted within PBS + heparin. Red blood cells were lysed twice with 1X BD Pharm lysing solution. Labelling was carried out in PBS+1%BSA.
Instrument	BD LSRII (4-laser, UV325, V405, B488, R640)
Software	BD FACSDIVA was used for acquisit and recording cells. Finally, gating, ploting were performed in flowjo 10.7.
Cell population abundance	Abundance of cell population gating were indicated figures. The purity of sorted cells were determined by flow cytometric analysis of sorted cells with the same gating strategy as during sorting. Cell populations were analyzed by gating CD45+Ly6G+ neutrophil. Depending on the experimental question, a fraction of FITC+ cells were calculated in neutrophil cluster. One of examples was plotted with numerical value of percentage and a ridgeline plot summarized FITC cell distribution in all groups.
Gating strategy	Intact cells were gated based FSC-A and SSC-A. We removed doublets by FSC-A and FSC-H, SSC-A and SSC-H. LIVE/DEAD fixable near-IR dead cell staining kit was used to exclude dead cells. Neutrophil was gated by CD45-APC and Ly6g-PB.

☒ Tick this box to confirm that a figure exemplifying the gating strategy is provided in the Supplementary Information.
