# Supplementary figures and images for: Neutrophils direct preexisting matrix to initiate repair in damaged tissues
Source: Nat Immunol. 2022 Mar 30;23(4):518–31. doi: 10.1038/s41590-022-01166-6 (PMC8986538; doi:10.1038/s41590-022-01166-6)

GAPDH

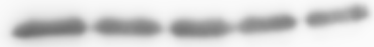

Kindlin3

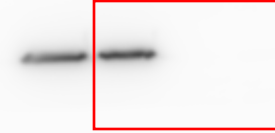

GAPDH

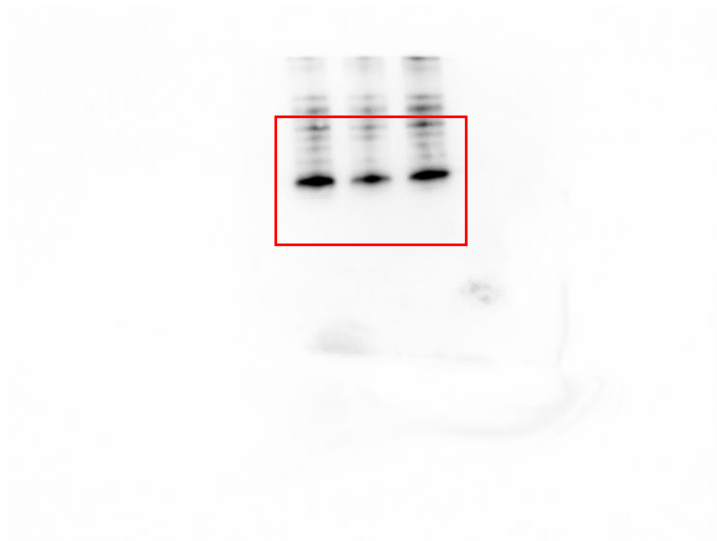

pHSF

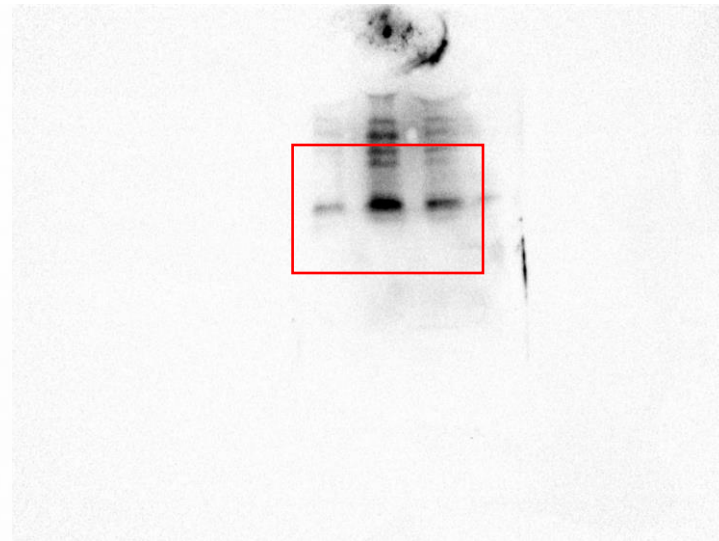

rat

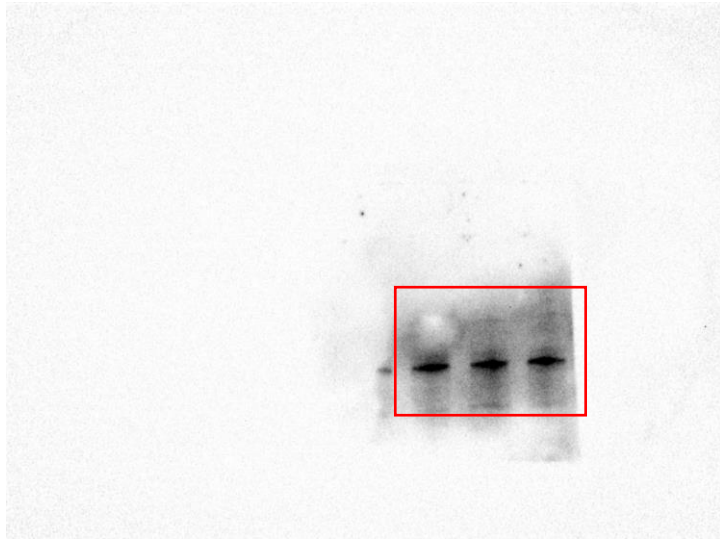

ITGAM

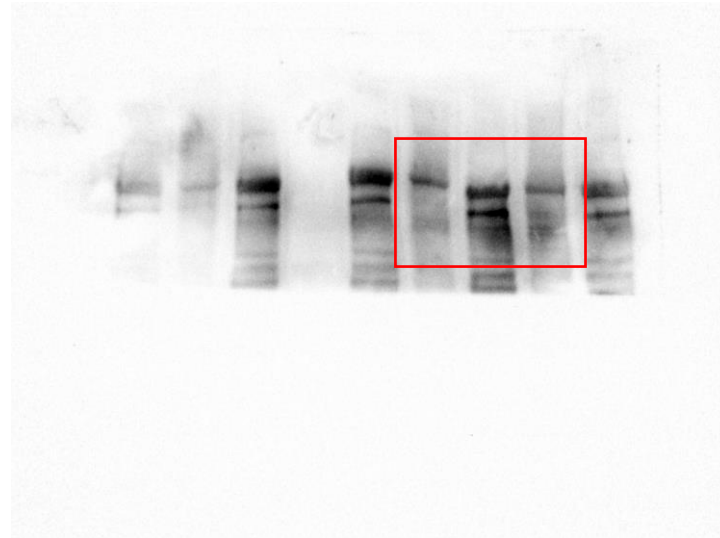

ITGb2

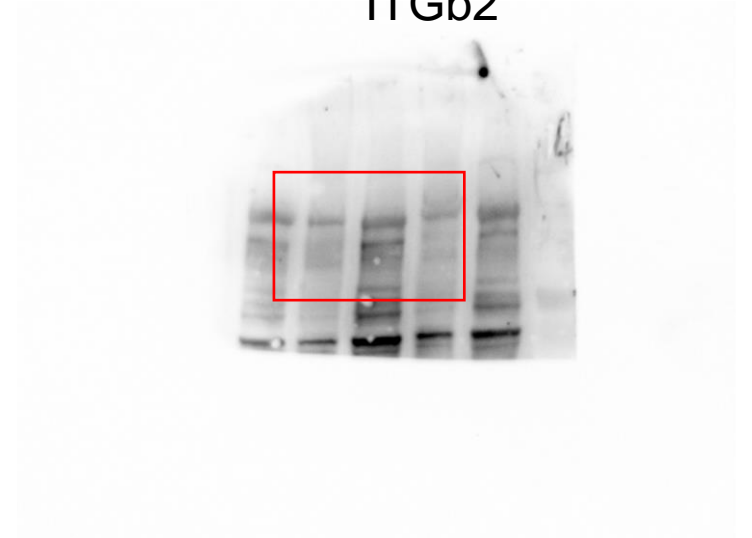

Supplement: Source Data Extended Data Fig. 8 — Unprocessed Western Blots [file 41590_2022_1166_MOESM28_ESM.pdf]
